# Demystifying Volume Status: An Ultrasound-Guided Physiologic Framework

**DOI:** 10.1016/j.chest.2024.12.026

**Published:** 2025-01-07

**Authors:** Juliana YL Kan, Shane Arishenkoff, Katie Wiskar

**Affiliations:** a Department of Internal Medicine, Singapore General Hospital, Singapore, Republic of Singapore; b Department of Medicine, University of British Columbia, Vancouver, BC, Canada

**Keywords:** fluid status, hemodynamics, physiology, point-of-care ultrasound, volume status

## Abstract

**Topic Importance:**

Accurate assessment of a patient’s volume status is crucial in many conditions, informing decisions on fluid prescribing, vasoactive agents, and decongestive therapies. Determining a patient’s volume status is challenging because of limitations in examination and investigations and the complexities of fluid homeostasis in disease states. Point-of-care ultrasound (POCUS) is useful in assessing hemodynamic parameters related to volume status, fluid responsiveness, and fluid tolerance. It requires understanding several physiologic concepts to interpret and integrate POCUS findings accurately into volume-related clinical decision-making.

**Review Findings:**

The following concepts serve as a scaffold for a comprehensive volume status assessment: central venous pressure, right-sided heart function, left-sided heart assessment, extravascular volume, and venous congestion. POCUS allows us access to these hemodynamic and structural data points as an extension and refinement of the physical examination. Often, multiple POCUS applications are used, and findings must be integrated with the rest of the clinical evaluation. We illustrate this using 3 common scenarios: hypotension, hypoxia, and acute kidney injury. Clinicians must be aware of the strengths and weaknesses of findings in different physiologic states and the potential pitfalls of image acquisition and interpretation. Further studies are necessary to determine the benefits and clinical outcomes of a POCUS-directed volume status assessment.

**Summary:**

Volume status assessment is ubiquitous, yet is challenging to perform. This review summarizes foundational physiologic concepts relevant to volume status evaluation and highlights how multiorgan POCUS elucidates hemodynamic parameters that can be combined with the conventional clinical assessment to make fluid-related decisions.

Accurate assessment of a patient’s volume status is crucial in multiple clinical scenarios such as heart failure, kidney disease, cirrhosis, and sepsis.^1^, ^2^, ^3^, ^4^ With the end goal of optimizing organ perfusion, decisions on administration of fluids, vasoactive agents, and decongestive therapy rely heavily on an accurate volume status assessment.^5^ Although the notion of volume status is a familiar concept among physicians, the term remains nebulous. Classification using the terms *hypervolemia*, *euvolemia*, and *hypovolemia* lack the precision necessary to describe adequately the complex relationship between fluid compartments and hemodynamic factors in the cardiovascular circuit. Volume status is contextual and dynamic, rather than a uniform state with a fixed definition.

Unfortunately, a patient’s volume status is notoriously challenging to assess at the bedside, and no standardized list of findings exists that defines a volume status assessment.^6^^,^^7^ Techniques such as assessing the jugular venous pressure, lower extremity edema, and the hepatojugular reflex can be challenging, are limited by suboptimal test characteristics, and are not direct measures of volume; rather, these are hemodynamic factors that can be altered independent of volume.^5^^,^^8^, ^9^, ^10^ Available laboratory investigations commonly associated with volume overload such as serum brain natriuretic peptide levels have limited specificity.^11^ Furthermore, much of the cardiovascular circuit is not accessible to examination, forcing clinicians to make inferences about volume based on an incomplete data set.

Adding to the difficulty of a volume status assessment is the complexity of the cardiovascular circuit itself. This does not behave anatomically or functionally as a simple uniform compartment, with intravascular blood volume unequally distributed among the arterial and venous systems and significant volumes stored in venous reservoirs.^12^ Regional differences in compliance, resistance, and capacitance render it near impossible to draw generalized conclusions based on findings in a single compartment. Increasing attention also is being paid to the potential harms associated with venous congestion and the need to balance fluid responsiveness (forward flow) with fluid tolerance (back pressures from the venous system). Furthermore, hemodynamic congestion may or may not be associated with systemic fluid overload, again underscoring the distinction between pressure and volume. Overall, accurate assessment of volume status is fraught with difficulties, and misinterpretations may lead to errors in management.^13^^,^^14^

Point-of-care ultrasound (POCUS) is a bedside ultrasound examination performed as an extension of the physical examination to help guide clinical management and can be useful in assessing volume status.^8^^,^^15^ POCUS allows for evaluation of physiologic and hemodynamic parameters related to volume status, fluid responsiveness, and fluid tolerance.^16^ Used in combination with other clinical parameters, ultrasound examination of the heart, lungs, and vasculature can help to answer complex volume-related questions and is particularly helpful in challenging physiologic situations such as cirrhosis, kidney disease, and cardiorenal syndrome.^5^^,^^16^, ^17^, ^18^, ^19^

However, like any tool in medicine, POCUS has pitfalls and caveats. POCUS-derived data points are linked inextricably to the individual patient’s physiologic characteristics, and failure to consider the impact of important anatomic and hemodynamic-related factors leads to misinterpretation. An understanding of several fundamental physiologic concepts is imperative for physicians who are integrating POCUS findings in volume-related clinical decision-making: (1) central venous pressure, (2) right-sided heart function, (3) left-sided heart assessment, (4) extravascular volume, and (5) venous congestion. This narrative review aimed to clarify these concepts and their role in performing comprehensive ultrasound-assisted volume status assessments, including a discussion of the strengths and weaknesses of POCUS findings in different physiologic states. This article presumes a basic understanding of POCUS applications. Discussion of image acquisition techniques and their challenges are beyond the scope of this review and are well described elsewhere.^20^

## Literature Review

This review draws on seminal works known to the authors related to ultrasound assessment of volume status and related physiologic concepts; citation searching was applied to draw in updated studies. In addition, we performed a targeted literature search of PubMed and Medline using keywords such as POCUS, ultrasound, volume status, central venous pressure, hemodynamics, and echocardiography. Papers considered by the authors to be most relevant to the concepts in this paper were included in the review.

## Evidence Review

### Central Venous Pressure

#### POCUS Findings

Central venous pressure (CVP) can be inferred from (1) the internal jugular vein (IJV)^21^, ^22^, ^23^, ^24^ and (2) the inferior vena cava (IVC) (Table 1).^25^, ^26^, ^27^ CVP is defined as the mean vena caval pressure or right atrial pressure, which, in the absence of tricuspid stenosis, is equal to the right ventricular end-diastolic pressure. Although widely discussed, several key aspects of the CVP often are understood poorly and bear clarifying.

**Table 1:** POCUS Applications in the Volume Status Assessment

Physiologic Concept	POCUS Application	POCUS Findings	Interpretation	Example	Caveats or Confounders
Central venous pressure	IJV	• The height of the JVP is taken as the point of maximal tenting of the IJV in the neck, measured in centimeters above the sternal angle. • Assess if IJV is ovoid or circular. • Assess the ratio of the IJV to CCA diameter.	An elevated JVP, a circular IJV, and a higher ratio of IJV to CCA diameter correlate with an elevated CVP (Video 1).		• Increased right atrial depth may lead to an underestimation of CVP if purely based on JVP height. • IJV stenosis or thrombus, SVC stenosis, pulmonary hypertension, and TR may to an elevated, distended, or circular IJV that is unrelated to volume.
	IVC	• Measure the diameter of the IVC. • Assess if the IVC is ovoid or circular. • Assess the respiratory variability of the IVC with a sniff test.	A distended, plethoric, spherical IVC with minimal inspiratory collapse corresponds with an elevated CVP in spontaneously breathing patients (Videos 2, 3).		• TR may result in a chronically distended IVC. • In cirrhosis, the IVC may be dilated because of portosystemic collaterals or may be constricted by a hypertrophied caudate lobe. • In the setting of raised intra-abdominal pressure, the IVC size may be underestimated.
Right-sided heart function	RV size and function	• Assess RV size. • Assess RV systolic function qualitatively or measure the TAPSE. • RV free-wall thickness.	• The RV is dilated (on qualitative assessment) when it is > 2/3 the size of the LV (Video 4). • Reduced TAPSE suggests reduced RV systolic function. • A dilated RV with reduced systolic function favors volume intolerance. • A thickened RV wall suggests chronic pulmonary hypertension.		• In the presence of LV dilation, RV dilation may be underestimated. • It is challenging to distinguish between acute and chronic right-sided heart failure.
	IVS	Identify flattening of IVS (ie, D-shaped septum).	Presence of D-shaped septum suggests RV volume or pressure overload (Video 5).		Volume and pressure overload may coexist.
	TR	Identify TR.	The presence of significant TR affects interpretation of CVP when making volume-related decisions (Video 6).		In RV failure, TR may be underestimated because of failure of RV to generate sufficient contractile forces.
	Pericardial space	Identify pericardial effusion and its impact on RA and RV filling.	The presence of RA or RV collapse, or both, during diastole suggests compromised right-sided heart filling and tamponade physiologic feature (Video 7).		Hemodynamic effect of pericardial effusions are related to the volume, rate of accumulation, and patient’s intravascular volume status.
Left-sided heart assessment	LAP	Assess MV inflow velocity (E), and mitral annular excursion (e′).	• E/e′ > 14 suggests elevated LAP and can be seen in impaired diastolic function. • An elevated LAP favors volume intolerance.		E/e′ measurements are affected by intrinsic mitral valve pathologic features.
	LV systolic function	LV systolic function can be estimated visually.	• Reduced myocardial thickening, myocardial excursion, and chamber emptying suggest reduced LV systolic function (Video 8). • A hyperdynamic LV may suggest intravascular hypovolemia or reduced SVR in the appropriate clinical context (Video 9).		• Recall that EF is not always congruent with CO, and the latter is much more reflective of the patient’s current hemodynamic milieu. • Valve disease such as MR can confound EF assessment further.
	LV diastolic function	• Assess LA size. • Assess LV thickness.	• The LA is dilated (on qualitative assessment) when it is larger than the diameter of the RVOT and the aortic outflow tract in the parasternal long-axis view. • LVH is associated with an increased probability of diastolic dysfunction.		• LA dilation and LVH occur in chronically elevated LAP. In acutely elevated LAP (eg, sepsis), LA dilation and LVH may not be apparent. In such cases, E/e′ can be helpful. • Increased LV wall thickness on qualitative assessment via POCUS should trigger formal assessment for LVH.
	Calculate cardiac output	Obtain the LVOT diameter and use it to calculate the CSA of the LVOT. Obtain the LVOT VTI. Calculate SV by multiplying the LVOT CSA and the LVOT VTI.	SV is a measure of forward flow out of the LV. CO is calculated using SV multiplied by heart rate. Changes in SV can be monitored in response to fluid and decongestive therapies.		Consider that SV is affected by preload, contractility, and afterload.
Extravascular volume	Pulmonary edema	Assess distribution and quantity of B-lines in lungs.	• Bilateral symmetrical B-lines with a dependent gradient and smooth pleural line suggest cardiogenic pulmonary edema (Video 10).		B-lines may have inflammatory etiologies, such as pneumonia and interstitial lung disease.
	Pleural effusions	Assess quantity and character of fluid in pleural space.	• The presence of pulmonary edema and pleural effusions is associated with elevated LV filling pressures and the permeability of pulmonary vasculature (Video 11).		Pleural effusions, pericardial effusions, and ascites may have inflammatory causes, including infection and malignancy.
			• Serial examinations allow semiquantitative measurements of the degree of pulmonary edema and monitoring of response to decongestive therapies.		
	Pericardial effusion	Assess quantity and character of fluid in pericardial space.	Pericardial effusions related to total body volume overload typically are circumferential and anechoic (Video 12).		Hemodynamic effect is related to the volume, rate of accumulation, and patient’s intravascular volume status.
	Ascites	Assess quantity and character of free fluid in peritoneal space.	Serial examinations allow for monitoring of response to decongestive therapies (Video 13).		Ascites may have inflammatory causes, including infection and malignancy.
	Subcutaneous edema	Assess for presence of subcutaneous edema.	Subcutaneous edema leads to a so-called cobblestone appearance. In total body volume overload, this accumulates in dependent regions of the body.		Subcutaneous edema also can be seen in inflammatory causes, such as cellulitis. These tend to be more localized.
Venous congestion	Hepatic vein waveform	Assess pattern of venous waveform, in particular the S-wave (systole) and D-wave (diastole).	• Venous congestion is suspected when the hepatic vein waveform demonstrates S-wave < D-wave or S-wave reversal, portal vein waveform demonstrates > 30% pulsatility, or intrarenal vein waveform is interrupted.		Waveforms may be affected by non-volume-related variables, including the presence of cirrhosis, tricuspid regurgitation, low BMI, and intrinsic renal pathologic features, as well as congestion resulting from primary pressure-related disorders.
	Portal vein waveform	Assess pattern of venous waveform for pulsatility.	• Serial examinations allow monitoring of response to treatment, because venous waveform abnormalities improve with decongestive therapy. This is particularly helpful in patients with chronic cardiac pathologic features in whom parameters such as CVP may never normalize despite adequate decongestion.		
	Intrarenal vein waveform	Assess pattern of venous waveform for interrupted flow.			

See corresponding videos in the Multimedia section of the online article. CCA = common carotid artery; CO = cardiac output; CSA = cross-sectional area; CVP = central venous pressure; EF = ejection fraction; IJV = internal jugular vein; IVC = inferior vena cava; IVS = interventricular septum; JVP = jugular venous pressure; LA = left atrium; LAP = left atrial pressure; LV = left ventricle; LVH = left ventricular hypertrophy; LVOT = left ventricle outflow tract; MR = mitral regurgitation; MV = mitral valve; POCUS = point-of-care ultrasound; RA = right atrium; RV = right ventricle; RVOT = right ventricle outflow tract; SVC = superior vena cava; SV = stroke volume; SVR = systemic vascular resistance; TAPSE = tricuspid annular plane systolic excursion; TR = tricuspid regurgitation; VTI = velocity time integral.

First, although volume affects pressure, one must not conflate the two. An increase in CVP may be secondary to a primary pressure-related disorder, a volume-related disorder, or both. Elevated CVP may be an indication of a number of non-volume-related pathologic features, as listed in Table 2.^28^ Although CVP is not itself a measure of volume, it provides information about the right side of the heart’s compliance and performance, which reflects its ability to accommodate volume.

**Table 2:** Causes of Elevated CVP That Do Not Reflect Total Body Volume Overload

Pathologic Feature	Example
Elevated juxtacardiac pressure impeding venous return	Cardiac tamponade Tension pneumothorax
RV outflow tract obstruction	Pulmonary stenosis
Elevated pulmonary vascular resistance	Pulmonary embolism ARDS
Intrinsic RV failure	RV infarct with RV systolic dysfunction
Acute valve failure	Damage to the tricuspid valve associated with infective endocarditis

RV = right ventricle.

Second, elevated CVP is a precursor to the development of clinically significant venous congestion. Venous return to the right side of the heart is governed by the pressure gradient between the right atrium and peripheral veins and by venous resistance.^12^^,^^29^ This pressure gradient is small, with peripheral venous pressure usually of 8 to 10 mm Hg and normal CVP ranging from 0 to 8 mm Hg.^30^^,^^31^ Therefore, as CVP increases, a requisite rise in peripheral venous pressures occurs to maintain right-sided heart filling.

Third, CVP is impacted by the peripheral venous system. The venous system is a low-pressure, high-capacitance system containing approximately 70% of intravascular circulating blood volume. Venous volume can be divided physiologically into stressed and unstressed volume. Under basal conditions, only about 30% of the intravascular blood volume is stressed volume; that is, it produces tension in the venous walls contributing to pressure in the vessels. The proportion of volume contained within the stressed and unstressed compartments varies depending on venous tone and is independent of total volume. The venous system can respond to physiologic demands by recruiting and de-recruiting volume from the unstressed compartment by means of sympathetic-mediated venous constriction, converting unstressed blood volume to stressed blood volume.^12^

#### Key Points for CVP

(1) CVP is influenced by nonvolume variables, physiologic conditions, and hemodynamic responses to disease states, rather than being a true reflection of whole-body volume or fluid responsiveness.^14^ (2) A nonelevated CVP is the normal physiologic state and does not necessarily equate to a need for additional fluids. (3) An elevated CVP may represent volume intolerance, the pathologic features listed in Table 2, or both. (4) When the IJV and IVC findings on POCUS are discordant, careful examination for caveats or confounders should take place (Table 1).^32^, ^33^, ^34^, ^35^

### Right-Sided Heart Function

#### POCUS Findings

POCUS findings include (1) right ventricle (RV) size and function, (2) interventricular septum flattening, (3) presence of tricuspid regurgitation, and (4) presence of pericardial effusion (Table 1). Pathologic features in the RV inherently compromise fluid tolerance; thus, an understanding of RV physiologic features and assessment is imperative when interpreting volume-related problems.

The right side of the heart is a thin-walled compliant chamber with the capacity to accommodate wide ranges of volume, allowing for increases in cardiac output (CO) while maintaining a low filling pressure and optimizing venous return.^28^^,^^29^ Beyond certain limits, RV dilation eventually is limited by the constraints of the pericardium. Additional volume loading past this point will lead to elevated RV diastolic pressure, resulting in an increased CVP, which limits venous return. Septal flattening and ventricular interdependence also will result in impaired left ventricular filling in cases of RV pressure or volume overload.^35^

In settings of chronically elevated RV afterload, such as pulmonary hypertension, the RV adapts to higher pressures over time with cardiomyocyte hypertrophy, which increases contractility and RV systolic pressure without chamber dilation or CVP elevation. As soon as contractile adaptations are overwhelmed, the RV dilates to preserve stroke volume (SV), leading to eventual impairment of RV function and right-sided heart failure.^36^ This stage often is associated with significant functional tricuspid regurgitation and chronically elevated filling pressures.

Finally, it is worth describing the impact of the pericardial space on RV filling in the absence of RV dilation. In cardiac tamponade and constrictive pericarditis, increased pericardial pressure compromises RV filling because of extrinsic forces, which reduces preload.^37^ This underscores the distinction between volume and pressure and demonstrates that RV filling can be affected by nonvolume variables.

#### Key Points for Right-Sided Heart Function

(1) RV dilation and poor systolic function may represent excessive RV preload, excessive RV afterload, or intrinsic RV failure. Extra volume is tolerated poorly. (2) In significant tricuspid regurgitation and chronic pulmonary hypertension, an elevated CVP may not represent a volume overload state.

### Left-Sided Heart Assessment

#### POCUS Findings

POCUS findings include (1) left atrial pressure (LAP), (2) LV systolic function, (3) LV diastolic function, and (4) calculated CO (Table 1).^38^, ^39^, ^40^, ^41^

##### Overview

The end goal of optimizing a patient’s volume status and hemodynamic parameters is to ensure adequate perfusion. The primary function of the LV is to provide sufficient CO to the organs. CO is dependent on heart rate and LV SV, which is governed by preload, contractility, and afterload.^42^ Intuitively, the left side of the heart can pump out only what it receives, so left-sided heart preload is dependent further on intravascular volume and right-sided heart output.

##### Left Atrial Pressure

The left atrium is a reservoir, conduit, and pump to modulate LV filling.^43^ LAP correlates with LV end-diastolic pressure; it is a surrogate for LV end-diastolic volume, and thus may be used to assess LV preload.^44^ Elevated LAP may be seen in LV systolic dysfunction, diastolic dysfunction, or both; left-sided valve pathologic features; or disease states such as sepsis and volume overload.^45^ Although it remains important not to conflate pressure and volume, in the setting of a volume assessment, elevated LAP should raise concerns about a patient who is intolerant of fluid and at risk of pulmonary congestion.^44^ Although commonly used as a surrogate of pulmonary congestion, it is important to recall that CVP is a right-sided parameter and that discordance between left and right sides may occur, rendering direct LAP assessment useful in certain scenarios.

##### LV Systolic Function

Reduced LV systolic function is encountered frequently in patients with chronic heart failure, or more acutely as a consequence of a systemic illness (ie, septic cardiomyopathy). LV ejection fraction (EF) may be estimated visually or may be calculated using (end-diastolic volume minus end-systolic volume)/(end diastolic volume).^46^ Although a useful global parameter, EF is less reflective of the patient’s current physiologic milieu compared with velocity-time integral-derived SV/CO, as discussed later herein. A patient with a dilated LV may show normal CO despite significant systolic dysfunction. Furthermore, EF may be confounded by valve lesions such as mitral regurgitation, leading to overestimation of forward flow.

##### LV Diastolic Function

Patients with isolated diastolic dysfunction similarly are at risk of complications related to cardiac decompensation and dysvolemia. Diastolic dysfunction may occur acutely in conditions such as sepsis or chronically in patients with predisposing comorbidities. Recognition of impaired myocardial relaxation and filling is important because the therapeutic window for optimal fluid management in these patients likely is narrower.^47^ Although a comprehensive diastolic assessment is beyond the skillset of casual POCUS users, clues to diastolic performance are available and should be incorporated into a holistic volume status assessment (Table 1).^48^

##### Cardiac Output

When considering the relationship between volume and CO, the following equation provides important clues about the physiologic features associated with forward flow:

meanarterialpressure(MAP)=CO×systemicvascularresistance(SVR)+CVP.

Fundamentally, the purpose of administering a fluid bolus is to increase SV.^49^ The aim of increasing SV is to improve CO, with the end goal of optimizing tissue perfusion. Changes in a patient’s SV, CO, or both after a passive leg raise or small fluid bolus can be helpful in determining fluid responsiveness, and serial measurements can help to monitor response to fluid, diuretic, and vasoactive therapies.^50^, ^51^, ^52^ However, decisions about fluid administration must look beyond fluid responsiveness alone. Fluid therapy impacts both the macrocirculation, which is concerned with the pulsatile pressure flow in large arteries, and the microcirculation.^53^ For example, in sepsis, microcirculatory dysfunction, capillary occlusion, and capillary leakage causing tissue edema are common. Although fluid administration may improve SV, such benefits may be eclipsed by worsening microcirculatory tamponade and increased venous back-pressure, decreasing tissue oxygenation.^54^ That is, patients may be both volume responsive and volume intolerant simultaneously. Therefore, although CO and maintenance of arterial pressure are crucial to perfusion, maximizing CO and extinguishing volume responsiveness should not be the sole goal of resuscitative efforts, and consideration must be given to the overall impact that therapeutic interventions have on both the macrocirculation and microcirculation. Finally, it is worth emphasizing that CO does not equal MAP, because SVR may vary widely in disease states. Because it is relatively straightforward to measure MAP, CO, and CVP at the bedside, these values can be used to make clinically useful inferences about a patient’s SVR.

#### Key Points for Left-Sided Heart Assessment

(1) In the presence of elevated LAP, caution must be exercised during fluid administration. (2) SV and CO measurement are useful in determining the cause of shock. (3) LV EF does not equate to CO; CO is a more useful dynamic variable to follow in acutely unwell patients. (4) SVR is an important aspect of the hemodynamic profile that impacts the interpretation of other volume-related data points.

### Extravascular Volume

#### POCUS Findings

POCUS findings include (1) pulmonary edema, (2) pleural effusion, (3) pericardial effusion, (4) ascites, and (5) subcutaneous edema (Table 1).^55^, ^56^, ^57^ Despite myths and traditional practice patterns to the contrary, the development of extravascular volume including tissue edema may or may not coexist with increased intravascular fluid volume. Deciphering the cause of extravascular fluid and its relationship to intravascular volume require attention to the following physiologic principles.

Accumulation of extravascular fluid is the result of an imbalance between capillary filtration and lymphatic reabsorption. Broadly speaking, increased capillary filtration is the result of an increased transcapillary hydrostatic pressure gradient, transcapillary oncotic pressure differences, and increased capillary permeability.^58^^,^^59^ Increased hydrostatic pressure may be the result of an increase in total intravascular volume, volume redistribution, or venous insufficiency. This is associated commonly with neurohormonal dysregulation in conditions such as heart failure and cirrhosis.^60^^,^^61^ Furthermore, the hydrostatic pressure gradient also may be impacted by reduced interstitial fluid pressure.^58^ Finally, a disruption to the endothelial glycocalyx, increase in capillary permeability, or both also can lead to increased movement of protein-rich fluid from intravascular locations to the interstitium.^59^^,^^62^

The primary mechanism for fluid and protein reabsorption from the extravascular space is through the lymphatic system. Problems with reabsorption may arise from lymphatic obstruction or dysfunction. Lymphatic obstruction may occur via compression because of mass effect from tumors, compartment syndrome, or resistance to thoracic duct outflow secondary to increased CVP, which limits return of fluid to the central veins. Lymphatic dysfunction may be secondary to valvular incompetence or a reduction in contractility of lymphatic smooth muscle cells, as seen in systemic inflammatory conditions.^58^ The above mechanisms of extravascular fluid accumulation demonstrate how tissue edema can occur without substantial increase in intravascular volume, pressure, or both.

Extravascular fluid can accumulate in tissues, including the lungs and abdominal viscera, and in body cavities, including the pleural, peritoneal, and pericardial spaces. In general, accumulation of extravascular fluid in multiple spaces is associated with a systemic process leading to total body volume overload, whereas regional edema may represent local disease processes or redistribution of volume from other compartments.

#### Key Points for Extravascular Volume

(1) Extravascular volume accumulation in multiple spaces suggests total body volume overload and volume intolerance. (2) Increased extravascular volume alone (ie, pedal edema) is not necessarily a reflection of elevated intravascular volume. (3) Extravascular fluid accumulation may be related to an increase in pressure, volume, redistribution of volume from 1 compartment to another, or a combination thereof.

### Venous Congestion

#### POCUS Findings

POCUS findings include (1) hepatic vein waveform, (2) portal vein waveform, and (3) intrarenal vein waveform (Table 1). In combination with the IVC, this is termed the *venous excess ultrasound* examination.^63^, ^64^, ^65^, ^66^, ^67^, ^68^

Instead of pursuing euvolemia as it pertains to absolute fluid volume, perfusion should be the hemodynamic focal point of the volume assessment. Historically, cardiovascular and critical care research have focused on improving perfusion by optimizing forward flow, prioritizing CO and MAP in hemodynamically unstable patients. Guidelines have focused on aggressive fluid administration as a cornerstone therapy, together with inotropic and vasopressor support. Until recently, little attention was afforded to the detrimental effects of elevated venous pressures and increase resistance to venous return.^69^^,^^70^ More recent studies have demonstrated clearly the negative consequences of venous congestion in shock, cardiorenal syndrome, and hepatorenal syndrome.^70^, ^71^, ^72^ Volume overload, systemic venous hypertension, and tissue edema have deleterious effects on multiple organ systems and are associated with increased mortality.^71^^,^^72^

**Figure 1:**
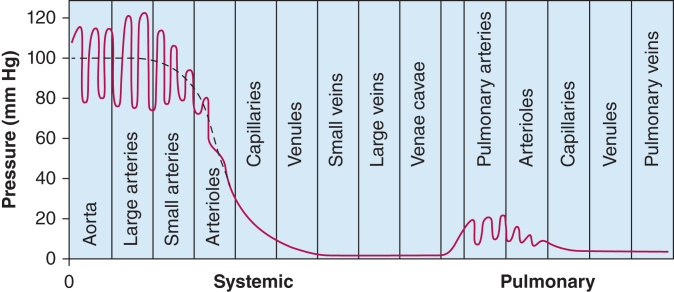
Graph showing normal BPs in the circulatory system when an individual is lying in the supine position. (Reprinted with permission from Guyton and Hall. *Textbook of Medical Physiology*. 12th ed. Elsevier; 2010:159*.*)^74^

Perfusion takes place at the level of the microcirculation, where the delta between capillary pressure and venous pressures is small (Fig 1). Excessive fluid administration leading to elevated venous pressures and reduced arteriovenous gradient across vital organs may hamper adequate perfusion.^63^ In addition, for encapsulated organs such as the kidneys, interstitial edema may cause a significant increase in interstitial pressure, tubular compression, and intracapsular tamponade, further reducing organ perfusion.^72^^,^^73^ Clinicians must consider the potential consequences of elevated venous pressures and compartment pressures when administering fluids to increase CO because a disproportionate rise in upstream venous pressure causing organ congestion may outweigh the benefits of an increased MAP.

Although elevated CVP identifies elevated right-sided heart filling pressures, this pressure may not necessarily be transmitted upstream, causing tissue congestion. Conversely, focal congestion can occur without an associated increase in CVP. Ultrasound evaluation of specific venous waveforms upstream from the central veins has been shown to correlate with congestion at the organ level and may provide additional specificity in this regard.^63^ However, as with CVP, changes in venous waveforms are a pressure phenomenon that may or may not be related to volume.

#### Key Points for Venous Congestion

(1) Fluid resuscitation end points not only should focus on MAP, but also consider the impact of venous congestion on tissue perfusion. (2) Abnormalities in venous waveforms suggest that organ impairment (congestive nephropathy and hepatopathy) may be secondary to venous congestion.^64^^,^^75^

## Clinical Applications

A physiology-based evaluation incorporating CVP, right-sided heart function, left-sided heart assessment, extravascular volume, and venous congestion serves as the scaffold for a comprehensive volume status assessment. POCUS allows us access to these hemodynamic and structural data points (Table 1). Because volume status is linked inextricably to an individual’s hemodynamic profile, the POCUS examination must account for the patient’s unique and often dynamic physiologic state. Findings must be integrated with a comprehensive clinical evaluation, including a review of relevant history and medications, clinical course including response to treatment, physical examination, laboratory results, and imaging investigations.^5^^,^^8^^,^^16^

The combination of POCUS applications most relevant to a volume-related problem depends on the clinical question at hand. We illustrate this using 3 common clinical scenarios: hypotension, hypoxia, and acute kidney injury (Table 3). Certain POCUS applications, such as assessing for other causes of hypoxia and for obstructive uropathy, are not covered in this article.

**Table 3:** POCUS Applications and Potential Findings in Clinical Scenarios

Clinical Scenario	Physiologic Concept	POCUS Interpretation
Shock (eg, cardiogenic: “cold and wet”)	CVP	Elevated JVP and plethoric IVC suggests elevated right-sided heart filling pressures.
	Right-sided heart function	RV dilation, reduced RV systolic function, interventricular septal flattening, and significant tricuspid regurgitation is consistent with RV dysfunction and with pressure or volume overload, or both.
	CO	Low CO suggests cardiogenic, hypovolemic, or obstructive shock.
		Passive leg raise shows absent fluid responsiveness.
	Extravascular volume	Pulmonary edema, pleural effusions, ascites, and subcutaneous edema suggest an excess in total body volume.
	Venous congestion	Abnormal hepatic, portal, and intrarenal venous waveforms suggest that elevated pressures are transmitted upstream.
Hypoxia (eg, flash pulmonary edema)	CVP	Nonelevated JVP and nonplethoric IVC are consistent with a normal CVP.
	LV filling pressure	Elevated LAP, dilated left atrium, and thickened LV myocardium.
	RV function	Normal RV size and systolic function suggests preserved RV function without volume or pressure overload.
	Extravascular volume	Diffuse, symmetrical pattern of B-lines in the lungs with gravitationally dependent gradient and smooth pleural line consistent with cardiogenic pulmonary edema. Bilateral pleural effusions.
	Venous congestion	Venous doppler not performed because CVP is not elevated.
AKI (eg, cardiorenal syndrome)	CVP	Elevated JVP and plethoric IVC.
	CO	Low CO suggests inadequate renal arterial perfusion pressure.
	Venous congestion	Interrupted intrarenal vein doppler, pulsatile portal vein, or hepatic vein S-wave reversal suggest venous congestion and likely congestive nephropathy.

AKI = acute kidney injury; CO = cardiac output; CVP = central venous pressure; IVC = inferior vena cava; JVP = jugular venous pressure; LAP = left atrial pressure; LV = left ventricle; POCUS = point-of-care ultrasound; RV = right ventricle.

Despite the multitude of additional data points accessible with POCUS, bedside evaluation of fluid in the cardiovascular system remains limited. Clinicians should avoid making assumptions about a patient’s intravascular volume status based on the findings of a single compartment. Using CVP as an example, a systematic review demonstrated a very poor relationship between CVP and blood volume, as well as the inability of CVP to predict fluid responsiveness in a wide spectrum of clinical conditions.^13^^,^^14^ This weak relationship of CVP as a surrogate of preload and volume responsiveness is the result of the unaccounted variables of vascular and cardiac compliance, nonvolume variables, and the unequal distribution of total body volume among the various cardiovascular compartments.^4^ Finally, an awareness of the potential caveats and pitfalls in image acquisition, interpretation, and clinical integration is paramount.

## Future Directions

As the number of POCUS applications expands and they become more advanced, the integration of multiorgan findings with a patient's unique physiologic features becomes increasingly complex, raising the risk of errors. Consequently, comprehensive training and oversight in POCUS training programs are essential. Research is needed to explore the clinical outcomes of POCUS-guided volume assessments in different disease states, supported by a systematic scanning protocol. These outcomes should be compared with existing noninvasive hemodynamic monitoring methods.

## Summary

Volume status is a ubiquitous yet nebulous clinical concept that frequently is oversimplified and addressed superficially in medical education. Unfortunately, an accurate volume status assessment remains elusive, despite traditional physical examination techniques. This narrative review summarizes foundational physiologic tenants in the evaluation of volume status and highlights how multiorgan POCUS can serve as a window into these hemodynamic parameters. In combination with conventional clinical assessment, these principles can help clinicians to make challenging fluid-related decisions across a wide range of pathophysiologic characteristics.

## Funding/Support

The authors have reported to *CHEST* that no funding was received for this study.

## Financial/Nonfinancial Disclosures

None declared.

## Supplementary Data

e-Online Data

e-Online Data

e-Online Data

e-Online Data

e-Online Data

e-Online Data

e-Online Data

e-Online Data

e-Online Data

e-Online Data
