# Supplementary material for: Demystifying Volume Status: An Ultrasound-Guided Physiologic Framework
Source: Chest. 2025 Jan 7;167(6):1667–83. doi: 10.1016/j.chest.2024.12.026 (PMC12202789; doi:10.1016/j.chest.2024.12.026)
Supplement: e-Online Data [file mmc10.docx]

**Video Legend (Online only)**

Video 1: Long-axis demonstration of the taper point of the internal jugular vein: the ultrasonographic jugular venous pressure (JVP).

Video 2: Long-axis view of a plethoric, non-varying inferior vena cava, consistent with an elevated central venous pressure (CVP).

Video 3: Short-axis view of a plethoric, spherical, non-varying inferior vena cava, consistent with an elevated central venous pressure (CVP).

Video 4: Apical 4-chamber view demonstrating a dilated right ventricle (RV) and right atrium with reduced RV systolic function.

Video 5: Parasternal short-axis view demonstrating septal flattening (“D-shaped septum”), consistent with right-sided overload.

Video 6: Right ventricle-centered apical 4-chamber view with Colour Doppler showing severe tricuspid regurgitation (TR).

Video 7: Subcostal 4-chamber view demonstrating a large pericardial effusion with evidence of chamber collapse.

Video 8: Parasternal long-axis view demonstrating reduced left ventricular (LV) systolic function.

Video 9: Parasternal short-axis view demonstrating hyperdynamic left ventricular (LV) function

Video 10: Lung ultrasound demonstrating vertical artifacts (B lines) with a smooth pleural line, in keeping with pulmonary edema.

Video 11: Lung ultrasound demonstrating extravascular fluid in the pleural space (pleural effusion).

Video 12: Parasternal short axis view demonstrating a circumferential pericardial effusion.

Video 13: Abdominal ultrasound demonstrating extravascular fluid in the peritoneal space (ascites).
